# Impact of Inflow Ratio on a Double-Inflow Cavopulmonary Assist Device

**DOI:** 10.1097/MAT.0000000000002645

**Published:** 2026-01-23

**Authors:** Simon Klocker, Leon Ballabani, Pascal Schmidt, Benjamin Torner, Bente Thamsen, Michael Röhrich, Michael Hübler, Daniel Zimpfer, Marcus Granegger

**Affiliations:** From the * Department of Cardiac and Thoracic Aortic Surgery, Medical University of Vienna, Austria; † Department of Anesthesia, Critical Care and Pain Therapy, Medical University of Vienna, Austria; ‡ Department of Congenital and Pediatric Heart Surgery, Children’s Heart Clinic, University Heart & Vascular Center Hamburg, Hamburg, Germany.

**Keywords:** cavopulmonary assist device, failing Fontan, flow estimator, inflow ratio, rotodynamic blood pump

## Abstract

The Fontan circulation is a palliative treatment for univentricular heart disease but is prone to progressive hemodynamic failure. To address this, a novel cavopulmonary assist device (CPAD) with dual inlets from the superior and inferior caval veins was developed. This study examines how varying inflow ratios (IRs) affect the CPAD’s hydraulic performance, hemocompatibility, and flow estimation accuracy. Hydraulic performance, represented by the pressure head-flow (H–Q) characteristics, was experimentally and numerically assessed at different IRs. Hemolysis was evaluated experimentally at the nominal operating point (4 L/min, 2,500 RPM) for IRs of 1:1 and 1:3 (n = 5). Additionally, hemocompatibility-related metrics were determined numerically. Furthermore, the robustness of conventional flow estimation methods, based on motor current, pump speed, and viscosity, under varying IRs was examined. *In vitro* and *in silico* results indicated low variations in both hydraulic performance (ΔH < 2.2 mm Hg) and hemolysis(22.4% in measured Normalized Index of Hemolysis [NIH]; 4.9% in predicted damage index [DI]) across all investigated IRs. The flow estimation model based on motor current, rotational speed, and fluid viscosity showed high accuracy regardless of the IR, with root mean square error (RMSE) less than 0.148 L/min and *R*² greater than 0.99. The analyzed double-inflow CPAD performed reliably across the investigated IRs, supporting its suitability for a broad patient population and enabling precise flow monitoring.

## Graphical abstract

**Figure:**
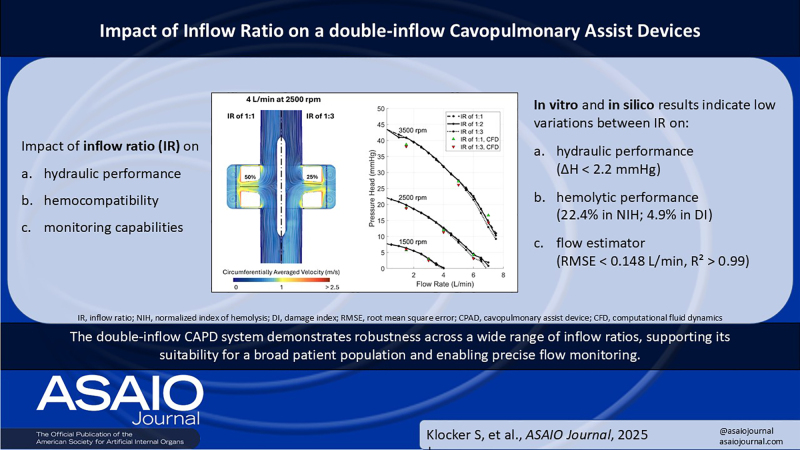

The failing Fontan circulation represents a significant and increasingly prevalent clinical challenge, as a growing number of patients encounter progressive complications following Fontan palliation.^1–3^ The Fontan procedure, initially effective in providing palliation for single-ventricle congenital heart disease, is associated with long-term morbidity and mortality.^3^ Among the various modes of Fontan failure, subpulmonary failure may account for a substantial proportion of cases.^4^ Although left ventricular assist devices (LVADs) have advanced significantly in the treatment of ventricular failure, therapeutic options for subpulmonary support are currently limited to temporary measures, underscoring a critical unmet need in this patient population.^5–7^

The defining feature of the Fontan circulation is the absence of a subpulmonary ventricle.^8^ This physiology imposes persistent strain on the cardiovascular system, frequently resulting in elevated systemic venous pressures, diminished cardiac output, and progressive systemic organ dysfunction.^1^ To counteract the lack of a subpulmonary ventricle, we previously developed an extravascular pump designed for implantation in the total cavopulmonary connection.^9^ This device demonstrated feasibility as a long-term cavopulmonary assist device (CPAD) capable of restoring subpulmonary support, alleviating systemic venous congestion, and improving hemodynamics.^10,11^

The double-inflow configuration of CPADs introduces additional complexities compared to LVADs. In particular, the inflow ratio (IR) between the two inlets can vary significantly due to the heterogeneity of the Fontan population (patient age, hemodynamic status) and levels of physical activity.^12,13^ These variations may impact the device’s hydraulic performance and hemocompatibility by introducing additional flow losses. Ensuring robustness across various IRs is critical for the success of addressing the heterogeneity of Fontan patients.^12^

Furthermore, a critical requirement for transitioning to chronic animal studies is the development of a reliable flow monitoring system. Positioned in series with the cardiovascular system, the device has a direct impact on venous return and cardiac output, necessitating precise real-time monitoring of flow rate to ensure safe and effective operation. Variations in IRs cannot be detected without additional sensors; therefore, a reliable flow estimator robust against IR variations is a prerequisite.

In conventional LVADs, flow is estimated through indirect measurements of electrical parameters such as motor current.^14^ Whether a similar approach is also applicable to blood pumps with a double inflow and under a wide range of IRs has not yet been investigated. Thus, it is essential to evaluate the applicability of existing flow estimation methods to the double-inflow design or if novel monitoring techniques need to be developed.

This study aims to evaluate the impact of IR on the device’s hydraulic performance, hemocompatibility, and monitoring capabilities. The findings will further support progression to chronic animal models, ultimately contributing to the development of a durable, long-term cavopulmonary assist solution for subpulmonary failure in patients with failing Fontan circulation.

## Materials and Methods

**Figure 1.**
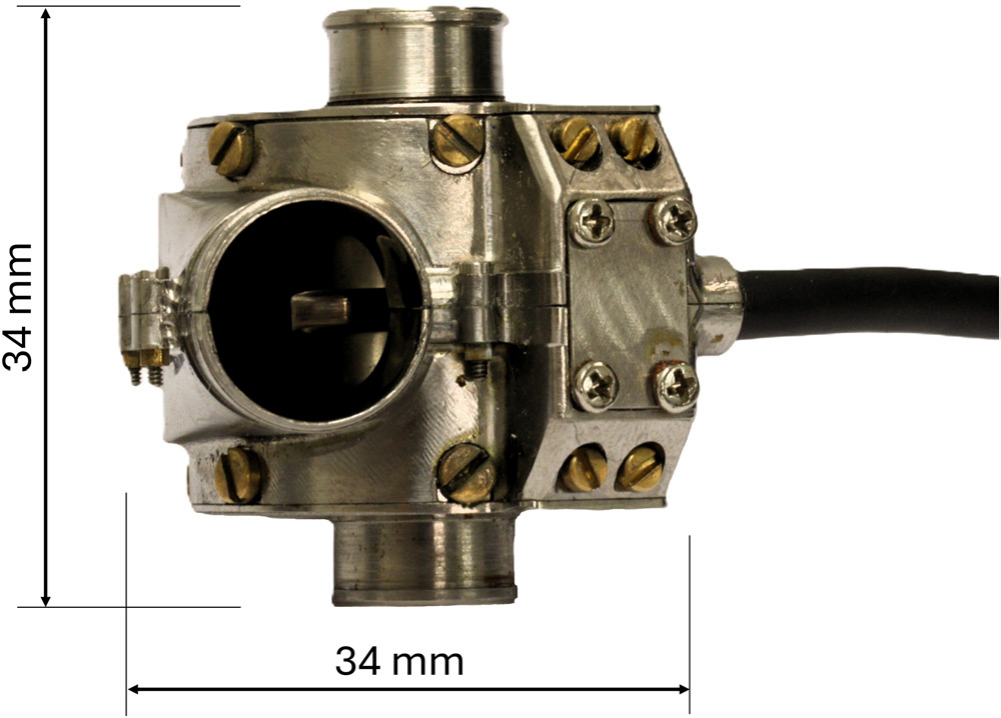
The second-generation CPAD prototype was used for *in vitro* assessment. The inlet from the SVC is positioned at the top, the inlet from the IVC at the bottom, and the single outflow is located at the front. CPAD, cavopulmonary assist device; IVC, inferior vena cava; SVC, superior vena cava.

The herein analyzed CPAD denotes the second generation of a rotodynamic blood pump concept.^10^ Fundamentally, a mechanically suspended centrifugal impeller drives incoming blood from the caval veins into the pulmonary arteries, guided by a volute casing and powered by an axial-flux three-phase synchronous motor configuration, see Figure 1. The CPAD was designed for a nominal operating point of 4 L/min against a pressure head of 12.5 mm Hg, which was achieved at a rotational speed of approximately 2,500 RPM. Compared to the first-generation, the second-generation was reconfigured into a double-inflow, single-outflow configuration. This change was motivated by the limitations of the initial double-outflow design, which hindered chronic implantation in an ovine animal model.^11^ Additionally, virtual fitting in computed tomographic datasets from Fontan patients revealed limited versatility, whereas the single-outflow configuration improves implantability and adaptability to diverse anatomies.^15^

### Effects of Inflow Ratio Variation

To evaluate the impact of variations in IR on hydraulic performance and hemocompatibility, both *in vitro* and *in silico* assessments were conducted. The IR is defined as the ratio of flow through the superior vena cava (SVC) to the flow through the inferior vena cava (IVC). The heterogeneity of the Fontan population is reflected in the wide variability of IRs. Unfavorable hemodynamic conditions tend to be associated with higher IRs, whereas IR generally decreases with patient age.^12^ Additionally, the venous return through the IVC increases during exercise; therefore, the IR decreases.^13^ To capture this variability, IRs of 1:1, 1:2, and 1:3 were investigated in this study. Of note, a single device was used for all *in vitro* experiments and was thoroughly cleaned between each use.

#### Pump operating conditions

The hydraulic performance was experimentally assessed at rotational speeds of 1,500, 2,500, and 3,500 RPM for IRs of 1:1, 1:2, and 1:3. Accompanied computational fluid dynamic (CFD) simulations—giving insight into the flow fields—have been conducted for the same rotational speed settings but limited to IRs of 1:1 and 1:3 to reduce computational cost. For each rotational speed, three flow rates were selected to cover the operating range.

Hemocompatibility was assessed numerically across the full operating range by considering hemocompatibility-related parameters in the CFD simulations. Numerical results were compared to *in vitro* hemolysis measurements for IRs of 1:1 and 1:3 at the design point (4 L/min, 2,500 RPM).

#### Computational fluid dynamics

The pump simulation setup was implemented in STAR CCM+ (Siemens Industries Digital Software, Plano, TX) and employed a polyhedral mesh containing 4.1 million cells. Impeller rotation was modeled using a sliding mesh, with a rotation of 4° per time step. The governing equations were solved in a segregated manner using the unsteady Reynolds-averaged Navier–Stokes (URANS) approach with the *k-ω SST* turbulence model.^16^ Temporal discretization was handled using a second-order scheme, and the segregated solver employed the SIMPLE algorithm.^17^ Normalized root mean square residuals dropped below 1 × 10⁻⁴ at each time step. Transient convergence was achieved after 20–50 revolutions, depending on the operating condition.

The CFD model was validated by comparing its predicted hydraulic performance, expressed through the pressure head *versus* flow rate (H–Q) characteristic, with corresponding experimental data.

In addition to the *in vitro* hydraulic performance assessment, CFD simulations provide qualitative insights into the mixing of flow from the two inlets. This was visualized using circumferentially averaged flow fields. Furthermore, CFD allows us to predict the inner pump efficiency, defined as


$$\eta \left(\%\right)=\frac{H\;Q}{T\;\omega }\times 100,$$ (1)

where H denotes the pressure head (Pa), Q the flow rate (m³/s), T the impeller torque (Nm), and ω the angular velocity (rad/s).

To evaluate the hemocompatibility, we analyzed volume exposed to specific shear stress thresholds (9, 50, and 150 Pa), volume of low velocity regions (*v* < 0.01 m/s), and the relative flow through the side chambers. The scalar shear stress $\tau_{s}$ was defined as


$$\tau_{s}=\mu \sqrt{2S_{ij}S_{ij}+\frac{1}{\nu }\epsilon_{turb}},$$ (2)

in which μ denotes the dynamic viscosity, $S_{ij}$ the strain rate tensor, ν the kinematic viscosity, and $\epsilon_{turb}$ the modeled turbulent dissipation rate.^18,19^ For all calculations, the time-averaged shear stresses, averaged over two impeller revolutions, were used. Furthermore, we calculated the damage index (DI) as a normalized fraction (0–1) based on the power law introduced by Giersiepen *et al*., using the empirical constants fitted to the experiments conducted by Heuser and Opitz (C = 1.8 × 10⁻⁶, α = 1.991, *β* = 0.765).^20–23^ The computed DI was time-averaged over two impeller revolutions. The same method was applied to the first generation for comparison in our analysis. Washout was modeled using an additional scalar transport equation and calculated for nominal load. Simulations were conducted using the same blood analog properties (1,110 kg/m³, 3.0 mPa·s) as in the hydraulic performance assessment.

#### Hydraulic performance assessment

**Figure 2.**
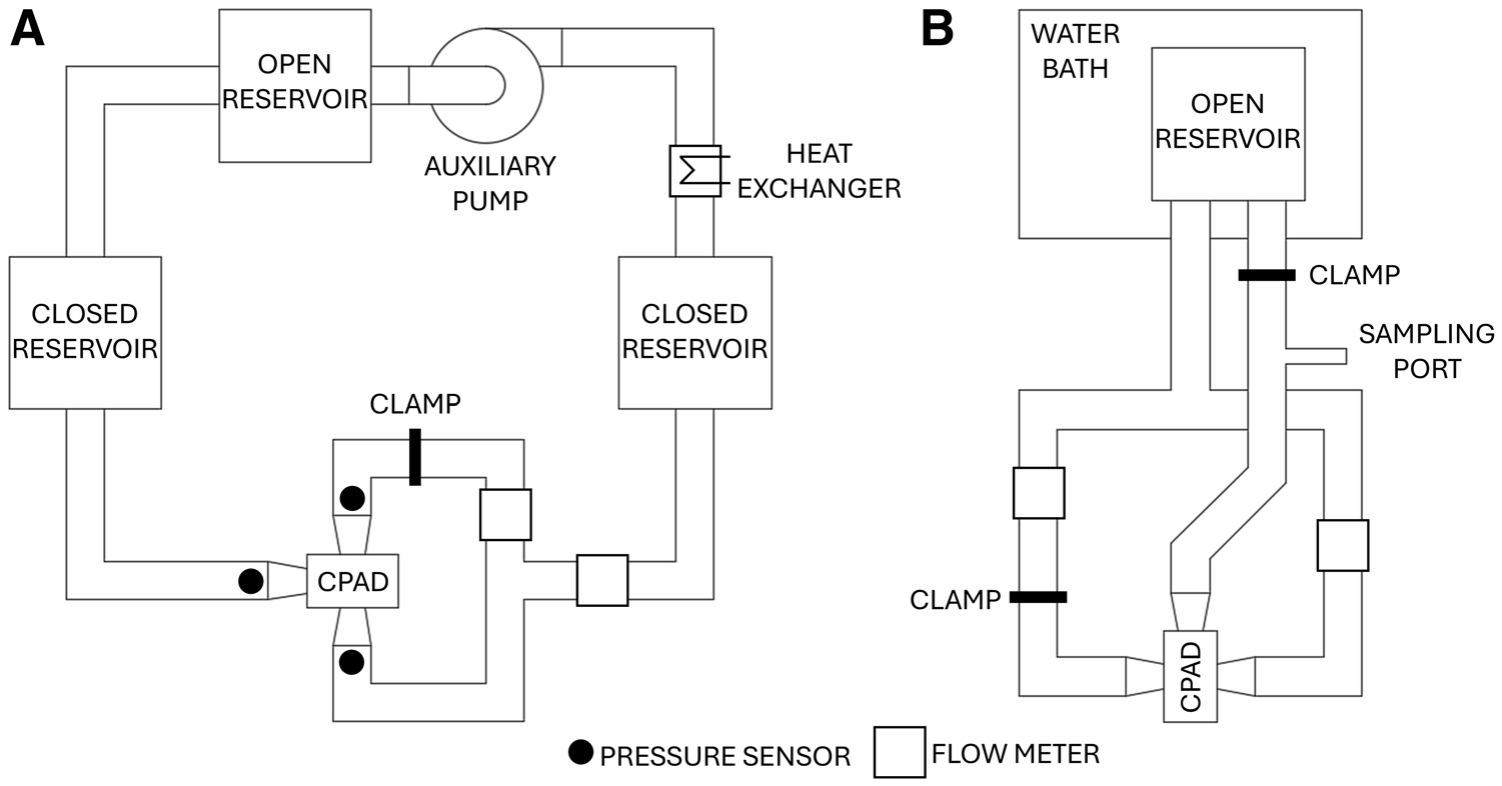
Experimental setups for (A) hydraulic assessment using a glycerol-water solution and (B) hemolysis evaluation using blood.

The experimental setup consisted of two closed reservoirs with the CPAD installed between them and an open reservoir positioned in front of an auxiliary pump, as illustrated in Figure 2A. The closed reservoirs served as a pressure buffer, while the open reservoir supplied the necessary fluid volume. The IR was adjusted using a proportional valve in the SVC pathway and was verified using two clamp-on ultrasonic flow meters (SONOFLOW CO.55, SONOTEC GmbH, Halle [Saale], Germany), one positioned before the branch and one in the SVC pathway. With the desired flow rate controlled by the auxiliary pump (UP3-R 24V, MARCO s.p.a., Brescia, Italy), the resulting pressure head at a set rotational speed was calculated as the difference between the total pressure at the outlet sensor and the averaged total pressure at the two inlet sensors. Total pressures were calculated during post-processing by adding the dynamic pressures to the static pressures from the measurements. To capture the complete H–Q characteristic at a given speed, the flow rate was gradually increased by 0.5 L/min increments until the measured pressure head became negative. This study was conducted using a glycerol-water solution (47% glycerol by weight, 1,110 kg/m^3^, 3.0 mPa·s) at a constant temperature of 37°C, maintained by a heat exchanger.

#### Hemocompatibility evaluation

The subsequent *in vitro* assessment of hemolysis was based on the ASTM International (ASTM) F1841-19 standard recommendations.^24^ We used heparinized bovine blood (600 ml, 15,000 IU/L, 35% hematocrit) at 37°C. Blood samples of 1.5 ml were collected every 30 minutes over a 3 hour period for each IR, resulting in a total experimental duration of 6 hours. The IR condition sequence varied across a total of five experiments. Sampling processing to derive plasma-free hemoglobin concentration is reported in the Supplemental Digital Content, https://links.lww.com/ASAIO/B788. Outliers were identified and subsequently removed using the interquartile range (IQR) method, with the lower and upper bounds defined as $Q_{1,3}\pm 1.5\;\;IQR$, where $Q_{1,3}$ denotes the first and third quartiles. Each experiment was analyzed using a linear regression model, where the slope of the fitted line represents the measured change in free hemoglobin concentration. We calculated the Normalized Index of Hemolysis (NIH) according to the ASTM F1841-97 standard and performed a paired t-test comparing both IR conditions (1:1 and 1:3).^24^

The experimental setup consisted of the CPAD connected to an open reservoir in a closed-loop system, as illustrated in Figure 2B. The IR was adjusted using a clamp in the SVC pathway and was verified with two clamp-on flow meters at the IVC and SVC pathways. An additional clamp, positioned downstream of the CPAD at the inlet to the open reservoir, regulated the total flow rate. A heated water bath (CORIO C, JULABO GmbH, Seelbach, Germany) surrounding the open reservoir maintained the temperature at 37°C. The sampling port was located downstream of the CPAD outlet.

### Development of a Flow Estimator

The flow estimator developed in this study is based on CPAD-related parameters, including motor current, pump rotational speed, and manual input of fluid viscosity. Such an approach is commonly used.^12^ Motor current at the IR of 1:1, 1:2, and 1:3 was measured during the hydraulic performance assessment. Additionally, to account for the influence of fluid viscosity, further measurements were carried out at IR 1:2 across a range of rotational speeds (1,500, 2,000, 2,500, 2,800, 3,000, and 3,500 RPM) and viscosities (2, 3, and 4 mPa·s). These viscosities correspond to hematocrit values of between 20% and 40%.^25^ The influence of IRs on motor current was quantified by calculating the root mean square error (RMSE) of the maximum deviation across all IRs.

Surface fitting tools in MATLAB (The MathWorks Inc., Natick, MA) were used to identify suitable equation structures for the relationship between motor current and flow rate for each viscosity. The resulting model was extended with additional terms to account for viscosity variations. The model coefficients were determined using the Nelder–Mead simplex algorithm, as implemented in the MATLAB function *fminsearch*.^26^ This optimization minimizes the RMSE between the estimated and measured flow rates. A 10-fold cross-validation was performed to ensure the model’s robustness. The statistical performance of the final model was evaluated using the coefficient of determination (*R*²) and RMSE. Additionally, the Bland–Altman method was employed to identify potential systematic biases.

## Results

### Effects of Inflow Ratio Variations

#### Hydraulic assessment

**Figure 3.**
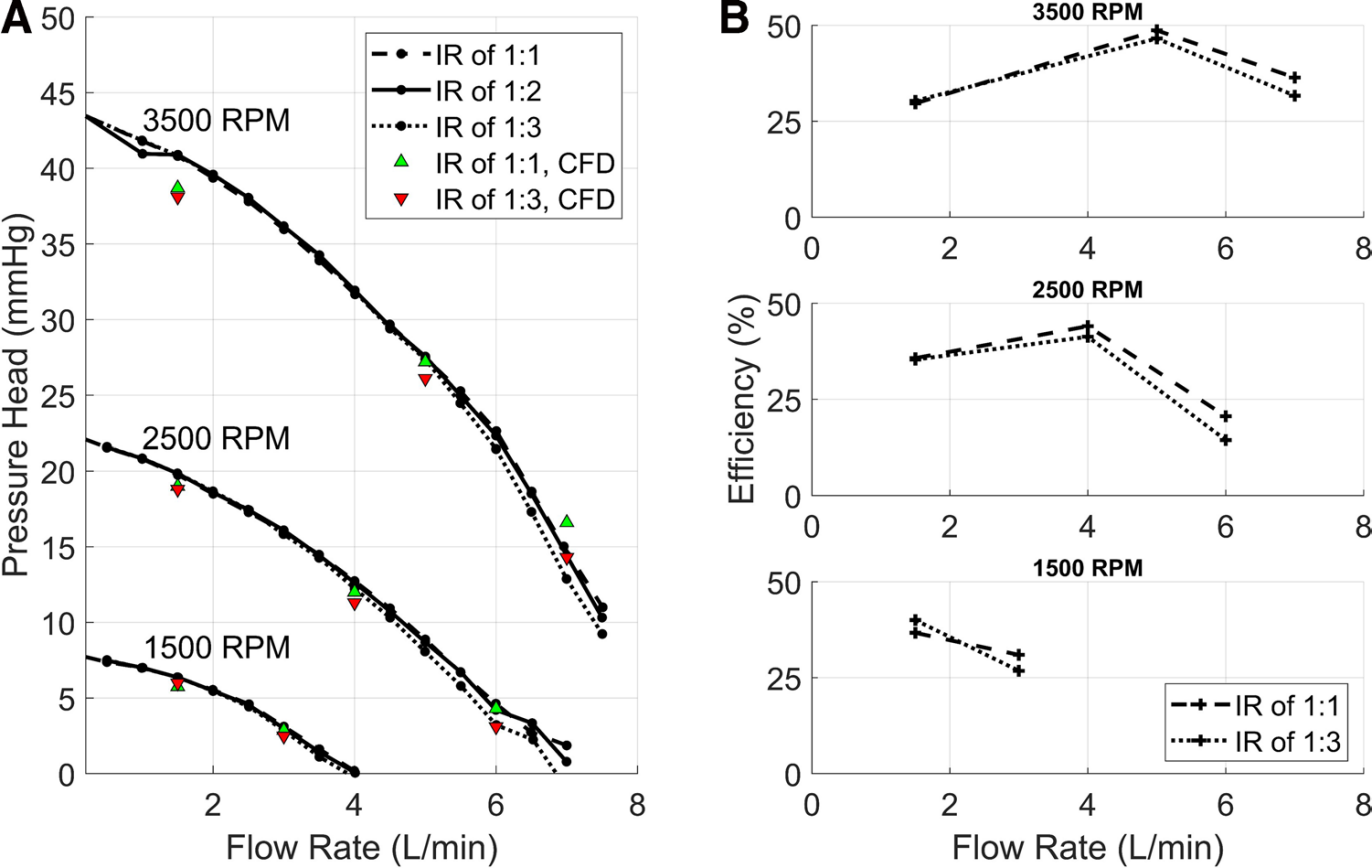
**Hydraulic assessment, A**: Measured characteristic pressure head *vs.* flow rate behavior at various IR and rotational speeds (1500, 2500 and 3500 RPM), including numerical results (CFD). The characteristics behaved largely unaffected by IR over a wide operating range, although deviations occurred at higher flow rates. **B**: Numerically calculated inner pump efficiency at various rotational speeds (1500, 2500 and 3500 RPM) showed similar trends, with increasing deviations at higher flow rates. CFD, computational fluid dynamics; IR, inflow ratios.

The experimentally assessed H–Q characteristics at the IRs of 1:1, 1:2, and 1:3 for three representative pump speeds (1,500, 2,500, and 3,500 RPM) are illustrated in Figure 3A. The results demonstrated a strong agreement between the IRs of 1:1 and 1:3, with an RMSE of less than 1.0 mm Hg. Although deviations were observed at higher flow rates, the discrepancy between IRs 1:1 and 1:3 remained within 2.2 mm Hg in all conditions.

The CFD results showed good agreement with the experimental data, with an RMSE of 1.0 mm Hg for IR 1:1 and 1.3 mm Hg for IR 1:3 (Figure 3A). Furthermore, the predicted H–Q curves exhibit the same overall trend as the measured H–Q curves, with deviations increasing at higher flow rates. Therefore, the numerical setup is considered globally validated.

The inner pump efficiency followed a similar trend, with higher deviations occurring at higher flow rates (Figure 3B). Across all the conditions investigated, the RMSE between the predicted efficiencies of the two IRs was 3.6%, with the maximum absolute deviation reaching 6.2%.

**Figure 4.**
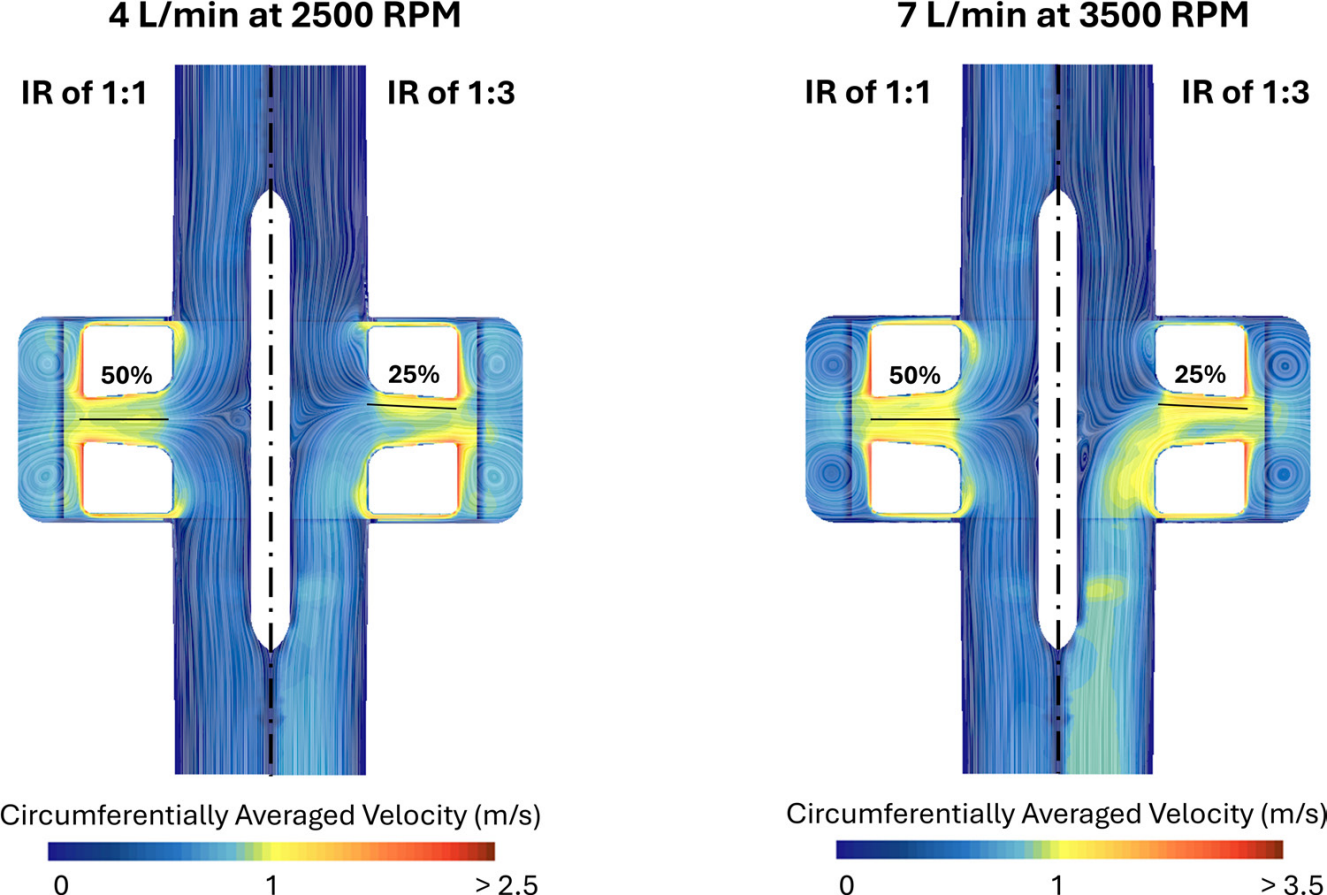
Circumferentially averaged absolute velocity fields at nominal (4 L/min, 2,500 RPM) and overload (7 L/min, 3,500 RPM). Markers indicate 50% (IR of 1:1) and 25% (IR of 1:3) of blade width. The artifacts at the interface between the stationary and moving domains, visible as low velocity areas, are a result of the circumferential averaging. These artifacts do not appear in the instantaneous flow field. IR, inflow ratios.

To visualize the inflow behavior, Figure 4 presents the circumferentially averaged flow fields in a mirrored, meridional cut-plane for both IRs at nominal load and at the investigated operation point with the highest deviation in pressure head (4 L/min, 3,500 RPM). At an IR of 1:1, the absolute velocity fields exhibited symmetrical characteristics, with the merging of the two inflows occurring at the midpoint of the blade width. To aid interpretation, reference lines at 50% and 25% of the blade width are included and correspond with IR of 1:1 and 1:3, respectively. In contrast, at an IR of 1:3, the merging regions shifted toward the SVC side, settling at approximately 25% of the blade width. These shifts are consistent with the IR, as an IR of 1:3 indicates that the SVC contributes 25% of the total inflow.

#### Hemocompatibility evaluation

The numerical results include the volume exposed to specific shear stress levels, the DI, the volume of low velocity regions, and the relative flow rate through the side chambers.

The values and corresponding relative deviations between the two investigated IR are reported in Table 1. The relative deviations in DI and in the volume exposed to shear stresses above 9 Pa are both below 10%. Notably, for other parameters, relative deviations can reach up to 30%; however, their change in absolute magnitudes remains below 1%, and in most cases, even lower. The time required for 95% washout of the pump volume under nominal load was 0.218 s at IR of 1:1 and 0.222 s at 1:3, resulting in a relative deviation of 1.8%. Finally, we compared the DI of the second-generation at nominal load (DI = 2.23–2.34 × 10⁻⁵ mg/100 L) with that of the first-generation (DI = 2.44 × 10⁻⁵) using the same method.

**Table 1.**
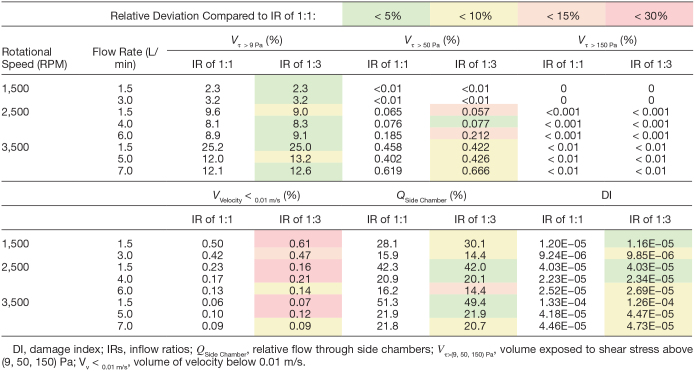
Simulated hemocompatibility-related metrics at IRs of 1:1 and 1:3

**Figure 5.**
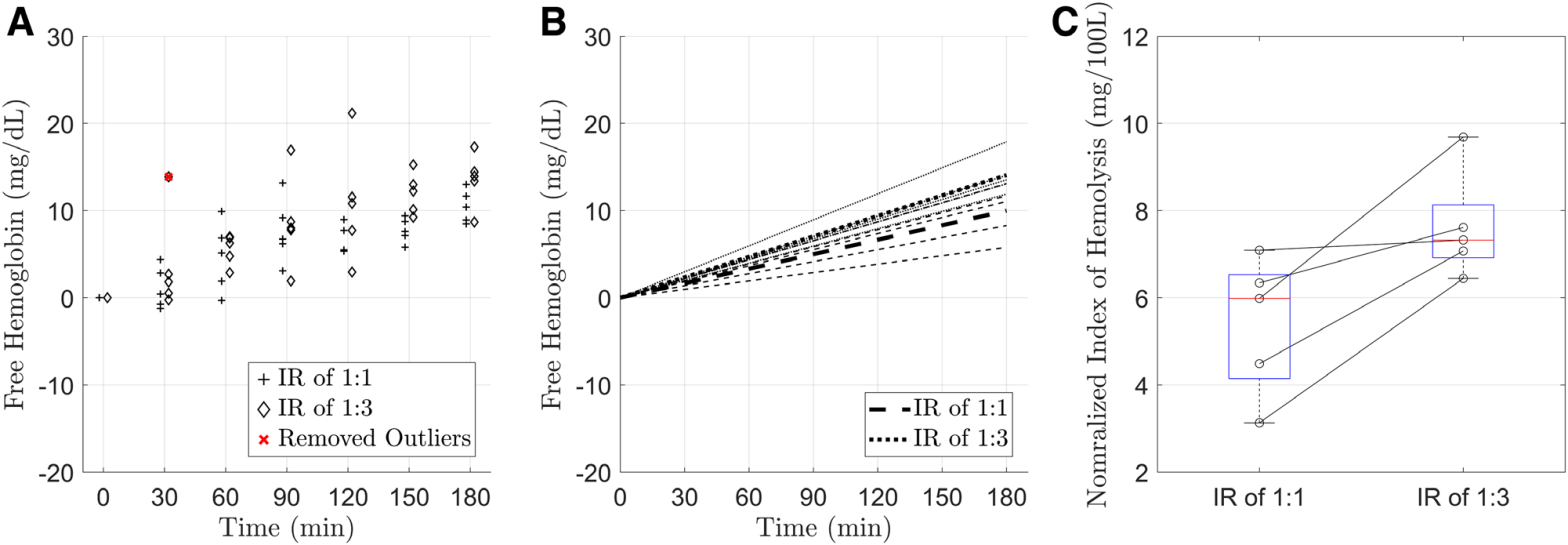
Hemolysis evaluation, **A**: Free hemoglobin concentration measured over time, including removed outliers. **B**: Linear regression analysis of free hemoglobin concentration over time for each experiment. **C**: NIH calculated according to the ASTM standard.^24^ IR, inflow ratio; NIH, Normalized Index of Hemolysis.

The experimentally assessed NIH was 5.98 mg/100 L at an IR of 1:1 and 7.32 mg/100 L at 1:3 (*p* = 0.026, paired t-test). Figure 5 presents the corresponding scatter plot of the measured free hemoglobin concentrations over 3 hours (Figure 5A), the linear regression models for each experiment (Figure 5B), and the resulting NIH values (Figure 5C). Overall, hemolysis increased significantly after 3 hours. The *R*² values of the linear regression models considering all experiments for each condition were 0.90 for IR of 1:1 and 0.97 for 1:3.

### Flow Estimator

**Figure 6.**
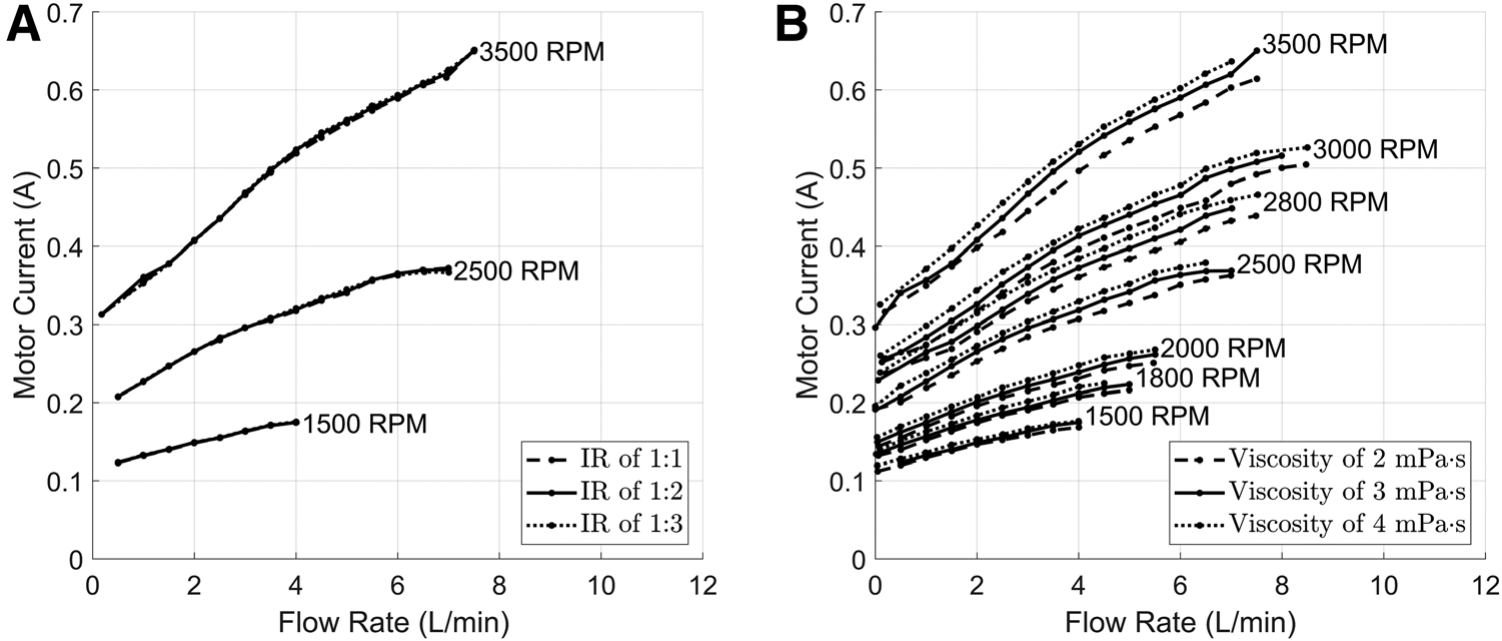
The influence of (**A**) inflow ratio (IR), and (**B**) fluid viscosity on the relationship between motor current and flow rate. Although IR shows no visible effect, increased viscosity leads to a rise in motor current. IR, inflow ratio.

Variations in IR had a minor impact on motor current, with a maximum RMSE of 0.0035 A observed across all IRs, as demonstrated at three rotational speeds in Figure 6A. In contrast, viscosity had a much stronger influence on motor current, with a maximum RMSE of 0.021 A, as illustrated in Figure 6B.

Curve fitting of the flow rate *versus* motor current behavior for each viscosity revealed that a cubic polynomial regression model provides a good fit. Consequently, a multivariate polynomial model incorporating viscosity-dependent terms is employed to estimate the flow rate $Q_{est}$, expressed as:


$$Q_{est}=\sum_{j=0}^{3}\sum_{k=0}^{3-j}\left[\left(A_{j,k}\cdot \mu +B_{j,k}\right)\cdot I^{\;j}\cdot \omega^{k}\right],$$ (3)

where I(A) denotes the motor current, μ(mPa·s)  the fluid viscosity, and $\omega \left(rad\cdot s^{-1}\right)$ the rotational speed. The identified coefficients $A_{j,k}$ and $B_{j,k}$ are provided in the Supplemental Digital Content, https://links.lww.com/ASAIO/B788.

**Figure 7.**
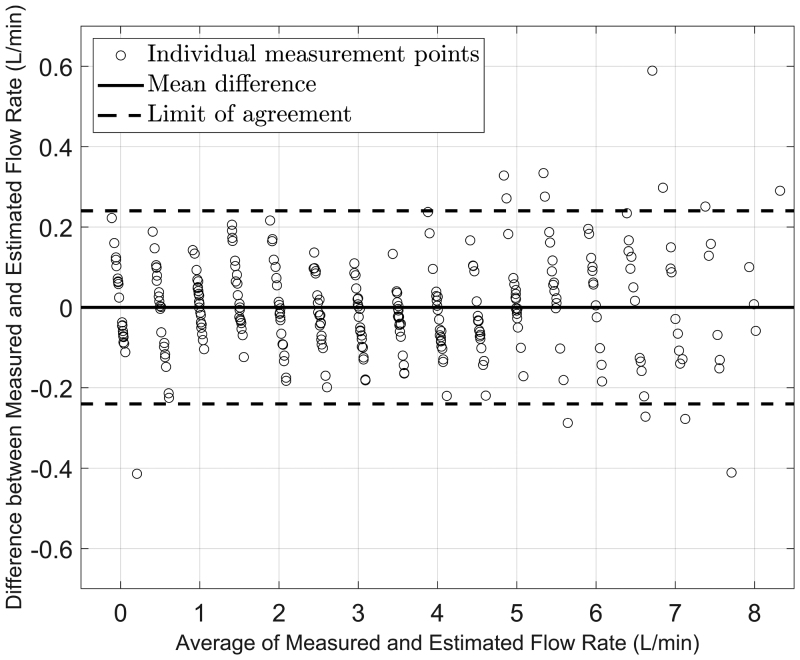
The Bland–Altman plot shows the difference between measured and estimated flow rates *vs.* their average. The limits of agreement are defined as ±1.96 standard deviations, representing the 95% range under the assumption of a normal distribution.

The resulting estimator achieved an RMSE below 0.148 L/min and a coefficient of determination *R*² above 0.99. Moreover, the absence of systematic bias in the estimation is confirmed across the evaluated range of total flow rates by the adapted Bland–Altman plot in Figure 7.

## Discussion

The aim of this study was to investigate the effect of varying IRs on hydraulic performance and hemocompatibility, as well as their impact on the monitoring accuracy of conventional flow estimators under such conditions.

In both *in silico* and *in vitro* studies, we demonstrated that the hydraulic performance and hemocompatibility of our second-generation CPAD remained reliable across the investigated IRs. The deviations in pressure head (< 2.2 mm Hg) and efficiency (< 6.2%) observed at higher flow rates seem not critical. The numerically predicted DI and the volume exposed to shear stresses above 9 Pa remain within 10% relative deviation. Although higher relative deviations are observed for other parameters, their absolute magnitudes are minimal, suggesting that these differences may not compromise safe operation.

This finding supports a key design objective: ensuring effective merging of cavitation inflows within the impeller eye before their entry into the blade channels. Consistency across a wide range of IRs is particularly important for the Fontan population, where the device must function under variable hemodynamic conditions influenced by factors such as patient age, clinical status, underlying etiology, and physical activity levels. Merging of the two inflows before they reach the impeller, also achieved in devices like the mini-FCAD, may offer a distinct advantage over CPAD designs that use either dual impellers or dual blade sets on a single hub.^27,28^ In such designs, each impeller generates a unique pressure head *versus* flow rate (H–Q) curve, resulting in different pressure heads and torque outputs depending on the different flow rates through each inlet. This discrepancy can lead to unequal inlet pressures at each impeller for the SVC and IVC inlets. Moreover, depending on the IR, one or both blade sets may operate under off-design conditions, even if the overall system operates near the nominal design operating condition, potentially leading to compromised hemocompatibility.^29^

In the *in vitro* experiments, hemolysis at an IR of 1:3 increased by 22.4% compared to an IR of 1:1. The discrepancy between the relative difference in DI/NIH between the IRs may be attributable to the additional resistance from the clamp used to adjust the IR.^30^ Notably, the experimentally assessed NIH for our second-generation CPAD was observed to be higher at the nominal operating point than that of its predecessor (NIH = 3.8 mg/100 L).^10^ However, these levels remain generally low and fall within the range reported for fully magnetically levitated, commercially available blood pumps (NIH < 10 mg/100 L).^31–34^ It is important to note that hemolysis testing was conducted on a single device for each generation; thus, the observed differences may stem from manufacturing tolerances or variations in bearing alignment. This limitation is highlighted by CFD results, which indicate even lower blood damage at nominal load for the second-generation CPAD (DI = 2.23–2.34 × 10⁻⁵) compared to the first-generation (DI = 2.44 × 10⁻⁵). Notably, this limitation is mainly present when comparing various devices and is mitigated when performing paired tests under different conditions on a single device, such as variations in IR in this study.

Furthermore, we demonstrated that accurate, sensor-less flow estimation is feasible in our CPAD by leveraging a monotonic relationship between motor current and flow rate. This relationship can be reliably modeled using polynomial functions and was robust across the investigated IRs. However, as fluid viscosity significantly influences motor current—higher viscosity increases current demand at a given flow rate, and *vice versa*—viscosity must be incorporated into the flow estimation model as a manual input. This approach is consistent with strategies employed in LVADs such as the HeartMate 3 (Abbott Laboratories, North Chicago, IL) and formerly used HVAD (Medtronic, Minneapolis, MN). The resulting accuracy of flow estimation is comparable to, or even surpasses, that reported for other circulatory assist devices.^14^

Accurate and reliable flow estimation is particularly critical for a CPAD, as the device operates in series with the cardiovascular system. Continuous monitoring is essential to allow for precise adjustment of pump output in response to hemodynamic changes. This ensures adequate cardiac output under varying physiological conditions. Importantly, this can be achieved without the need for additional implanted sensors. Moreover, accurate flow estimation serves as a foundational element for the development of future physiological control algorithms, which aim to automatically adjust hydraulic performance based on the patient’s real-time circulatory demands.^35^

Despite its accuracy, the system shares inherent limitations common to other sensor-less flow estimation methods. Specifically, it relies on accurate manual input of fluid viscosity, as deviations can significantly affect estimation accuracy. Additionally, the presence of abnormal mechanical loads, such as increased torque resulting from thrombus formation in the impeller region, can lead to erroneous flow rate estimations.^36^ These factors must be considered to maintain the reliability of the flow estimation system. Of note, the flow estimation was developed with a glycerol-water solution; therefore, results need to be confirmed with blood.

Collectively, these findings demonstrate that our updated CPAD offers stable hydraulic performance, reliable hemocompatibility, and accurate flow monitoring across the investigated IRs. Thereby, key performance benchmarks essential for chronic animal studies are met.

## Acknowledgments

The computational results have been achieved using the Austrian Scientific Computing (ASC) infrastructure.

## Supplementary Material
