# Supplementary material for: Impact of Inflow Ratio on a Double-Inflow Cavopulmonary Assist Device
Source: ASAIO J. 2026 Jan 23;72(8):676–83. doi: 10.1097/MAT.0000000000002645 (PMC13399723; doi:10.1097/MAT.0000000000002645)
Supplement: Supplementary file 1 [file mat-72-0676-s001.pdf]

Supplementary Materials

S1: In-vitro Assessment of Hemolysis

Plasma-free hemoglobin (fHb) was photometrically determined using a Photometer 4040_V5+ (Robert Riele GmbH & Co KG, Berlin, Germany) following a twofold sample centrifugation at 5600×g for 15 minutes for plasma isolation (Microfuge 22R Centrifuge, Beckman Coulter, Brea, CA, USA), and subsequent dilution of 100 mL of the plasma with 1000 mL of sodium carbonate.

S2: Identified parameters for Flow Estimator

$$A_{j,k} = \begin{bmatrix} A_{0,0} & \cdots & A_{0,3} \\ \vdots & \ddots & \vdots \\ A_{3,0} & \cdots & A_{3,3} \end{bmatrix} = \begin{bmatrix} 0.037 & -1.157 & 4.117 & -3.825 \\ 0.429 & -6.762 & 10.081 & \\ 3.153 & -9.017 & & \\ 2.762 & & & \end{bmatrix}$$

$$B_{j,k} = \begin{bmatrix} B_{0,0} & \cdots & B_{0,3} \\ \vdots & \ddots & \vdots \\ B_{3,0} & \cdots & B_{3,3} \end{bmatrix} = \begin{bmatrix} 0.339 & -2.153 & 2.824 & -1.035 \\ 6.276 & -12.330 & 5.763 & \\ -4.476 & -2.704 & & \\ 0.398 & & & \end{bmatrix}$$
